# Human UCB-MSCs treatment upon intraventricular hemorrhage contributes to attenuate hippocampal neuron loss and circuit damage through BDNF-CREB signaling

**DOI:** 10.1186/s13287-018-1052-5

**Published:** 2018-11-21

**Authors:** Hyo Rim Ko, So Yoon Ahn, Yun Sil Chang, Inwoo Hwang, Taegwan Yun, Dong Kyung Sung, Se In Sung, Won Soon Park, Jee-Yin Ahn

**Affiliations:** 1 Department of Molecular Cell Biology, Sungkyunkwan University School of Medicine, 2066, Seobu-ro, Jangan-gu, Suwon, 16419 South Korea; 2 Single Cell Network Research Center, Sungkyunkwan University School of Medicine, Suwon, 16419 South Korea; 3 Department of Pediatrics, Samsung Medical Center, Sungkyunkwan University School of Medicine, 81 Irwonro, Gangnam-gu, Seoul, 06351 South Korea; 4 Department of Health Sciences and Technology, SAIHST, Sungkyunkwan University, Seoul, South Korea; 5 Stem Cell and Regenerative Medicine Institute, Samsung Medical Center, Seoul, 06351 South Korea; 6 Samsung Biomedical Research Institute, Samsung Medical Center, Seoul, 06351 South Korea

**Keywords:** Intraventricular hemorrhage, Mesenchymal stem cells, BDNF, CREB, Hippocampus

## Abstract

**Background:**

Human umbilical cord blood-derived mesenchymal stem cells (hUCB-MSCs) have been shown to prevent brain damage and improve neurocognition following intraventricular hemorrhage (IVH). However, the molecular mechanisms underlying the effects of hUCB-MSCs are still elusive. Thus, as the hippocampus is essential for learning, memory, and cognitive functions and is intimately involved in the ventricular system, making it a potential site of IVH-induced injury, we determined the molecular basis of the effects of hUCB-derived MSCs on hippocampal neurogenesis and the recovery of hippocampal neural circuits after IVH in a rodent model.

**Methods:**

We inflicted severe IVH injury on postnatal day 4 (P4) in rats. After confirmation of successful induction of IVH using MRI (P5), intracerebroventricular administration of MSCs (ICV-MSC) was performed at 2 days post-injury (P6). For hippocampal synaptic determination, a rat entorhinal-hippocampus (EH) organotypic slice co-culture (OSC) was performed using day 3 post-IVH brains (P7) with or without ICV-MSCs. A similar strategy of experiments was applied to those rats receiving hUCB-MSC transfected with BDNF-Si-RNA for knockdown of BDNF or scrambled siRNA controls after IVH. The molecular mechanism of the MSCs effects on neurogenesis and the attenuation of neuron death was determined by evaluation of BDNF-TrkB-Akt-CREB signaling axis.

**Results:**

We showed that treatment with hUCB-MSCs attenuated neuronal loss and promoted neurogenesis in the hippocampus, an area highly vulnerable to IVH-induced brain injury. hUCB-MSCs activate BDNF-TrkB receptor signaling, eliciting intracellular activation of Akt and/or Erk and subsequent phosphorylation of CREB, which is responsible for promoting rat BDNF transcription. In addition to the beneficial effects of neuroprotection and neurogenesis, hUCB-MSCs also contribute to the restoration of impaired synaptic circuits in the hippocampus and improve neurocognitive functions in IVH-injured neonatal rat through BDNF-TrkB-CREB signaling axis activation.

**Conclusions:**

Our data suggest that hUCB-MSCs possess therapeutic potential for treating neuronal loss and neurocognitive dysfunction in IVH through the activation of intracellular TrkB-CREB signaling that is invoked by hUCB-MSC-secreted BDNF.

**Electronic supplementary material:**

The online version of this article (10.1186/s13287-018-1052-5) contains supplementary material, which is available to authorized users.

## Background

Intraventricular hemorrhage (IVH), a condition in which a germinal matrix hemorrhage ruptures via the ependymal to the lateral ventricle, is a common and serious disorder in premature infants [1]. IVH is often associated with high mortality and neurologic morbidities such as learning impairments, mental retardation, and seizures in survivors. About 12,000 premature infants develop IVH every year in the USA alone [2]. The incidence of IVH in very low birth weight infants (< 1500 g) has declined from 40–50% in the early 1980s to 20% in the late 1980s [3]. However, in the last two decades, the occurrence of IVH has remained stationary [4]. In extremely premature infants weighing 500–750 g, IVH occurs in about 45% of neonates [5]. Thus, IVH continues to be a major problem for premature infants in modern neonatal intensive care units worldwide. However, few effective treatments are currently available to attenuate brain damage after severe IVH in preterm infants. Therefore, the development of a new therapeutic modality to improve the outcome of this devastating disorder is quite important.

IVH is associated with neuronal degeneration and cognitive dysfunction [6, 7]. The hippocampus is known to be essential for learning, memory, and cognitive dysfunctions [8] and is intimately involved in the ventricular system, making it a potential site of IVH-induced injury. Although the hippocampus has been studied as a target for injury-induced neuronal degeneration in CA1 to CA4 regions and in the dentate gyrus, where newly generating neurons are created through hippocampal neurogenesis [9, 10], few studies have investigated hippocampal injury after IVH in neonatal animal models.

Cell-based therapy especially using mesenchymal stem cells (MSCs) has emerged as a promising strategy for treatment of neurologic disorders including ischemic stroke, hemorrhagic stroke, traumatic brain injury, and spinal cord injury [11–14]. Previously, we demonstrated that transplantation of human umbilical cord blood (hUCB)-derived MSCs substantially decreased IVH-induced cell death and improved motor skill learning ability in the newborn rat model of IVH [15–17]. Moreover, we also demonstrated that brain-derived neurotrophic factor (BDNF) secreted by transplanted MSCs is one of the critical paracrine factors playing a seminal role in attenuating IVH-induced cell death and improving behavioral learning dysfunctions [17]. However, the precise protective mechanisms of MSCs against IVH-induced brain injury and the specific molecular downstream pathways mediating the protection of BDNF secreted by MSCs, especially against hippocampal neuronal injury and impaired neurogenesis after IVH, have not been elucidated yet.

In the present study, we determined the effects of hUCB-derived MSCs on hippocampal neurogenesis and the recovery of hippocampal neural circuits after IVH in a rodent model. Moreover, we showed that BDNF, released by hUCB-MSCs, activated TrkB-CREB signaling and mediated neuronal survival and neurogenesis, enhancing rat BDNF transcription, whereas depletion of BDNF from hUCB-MSCs failed to exert these effects. Furthermore, our behavioral experiments demonstrated that treatment with hUCB-MSCs after IVH improved the performance in neurocognitive tasks known to be dependent on hippocampal neural circuits and neurogenesis, reflecting the recovery of synaptic function.

## Methods

### Cell preparation

hUCB-MSCs were provided by Medipost Co., Ltd., Seoul, Korea [6, 7, 9, 10]. BDNF and scrambled siRNAs were obtained from Santa Cruz Biotechnology (sc-42121, sc-37007, Santa Cruz, CA, USA). MSCs were transfected with siRNA oligonucleotides using Oligofectamine (Invitrogen, Carlsbad, CA, USA) according to the manufacturer’s instructions. From the MSCs, BDNF expression was successfully knock down and sustained for at least 48 h after transfection of BDNF siRNA.

### Animal model

All experimental protocols were approved by the Institutional Animal Care and Use Committee of Samsung Biomedical Research Institute, and the study followed institutional and National Institutes of Health guidelines for laboratory animal care. All animal procedures were performed in an Association for the Assessment and Accreditation of Laboratory Animal Care International (AAALAC)-accredited specific pathogen-free facility. Newborn Sprague-Dawley rats (Orient Co., Seoul, South Korea) were used. Dam rats were allowed free access to laboratory chow and water and were maintained in an alternating 12-h light/dark cycle with constant room temperature and humidity. In P4 rat pups, IVH was induced by intracerebroventricular injection of a total of 200 μl of fresh maternal whole blood using a stereotaxic frame as described previously [11]. At P5, baseline brain injury was confirmed with brain MRI as described previously [11–13]. Only severe IVH-induced rats were included. On P6, 1 × 10^5^ MSCs in 10 μl of normal saline or an identical volume of saline were injected intracerebroventricularly to the IVH rats. Follow-up brain MRI was performed at P32. We assessed and monitored the condition of rat pups twice per day (all of the experimental groups including negative control, IVH injury only, hUCB-MSC treatment after IVH injury, hUCB-MSC (scramble siRNA control) treatment after IVH injury, and hUCB-MSC (siRNA for BDNF) treatment after IVH injury), especially for 7 days after modeling and for comparison between groups. We used at least 15 rats per each experimental group. All procedures were performed under inhaled anesthesia using a mixture of isoflurane (Ifran®, Hana, Korea) and 2:1 nitrous oxide to oxygen.

### Antibodies

Anti-p-AKT (S473, cat. 4060s) and anti-p-Erk (cat. 9101s.) antibodies were acquired from Cell Signaling (Danvers, MA, USA). Anti-β-actin (cat. sc-47778), cleaved caspase 3 (cat. sc-2217), and anti-p-Trk (cat. 7996) antibodies were acquired from Santa Cruz Biotechnology (Dallas, TX, USA). Anti-GFAP (cat. ab72602), p-TrkB (cat. ab131483), cleaved caspase 3 (cat. ab2302), and anti-annexin V (cat. ab14196) antibodies were obtained from Abcam (Cambridge, MA, USA). Anti-mitochondria (cat. MAB1273) antibody was acquired from Millipore (Darmstadt, Germany). Anti-p-CREB (cat. MA5-11192) antibody was acquired from Thermo Scientific. Alexa Fluor 488 goat anti-mouse secondary antibodies were obtained from Molecular Probes (Eugene, OR, USA).

### Western blotting

The hippocampi of P7 and P9 rats were dissected, washed with 15 ml of PBS, and immediately added to lysis buffer (consisting of 50 mM Tris-Cl, pH 7.4, 150 mM NaCl, 1 mM EDTA, 0.5% Triton X-100, 1.5 mM Na3VO4, 50 mM sodium fluoride, 10 mM sodium pyrophosphate, 10 mM beta-glycerolphosphate, 1 mM PMSF, and protease cocktail). Hippocampal cell lysates were mixed with × 5 SDS sample buffer, boiled, and analyzed by immunoblotting. Protein levels were quantified by densitometry and normalized to actin (ImageJ software).

### Reverse transcriptase polymerase chain reaction

Brain samples were obtained from a rat IVH/MSC model. Total RNA was extracted with TAKARA miniBEST Universal RNA extraction Kit (TAKARA, Japan) according to the manufacturer’s instructions. The reverse transcription reaction was performed using a PrimeScript 1st strand cDNA Synthesis Kit (TAKARA, Japan). The RT-PCR primer sequences for rat BDNF were (forward) 5′-GTCCACGGACAAGGCAA-3′ (nucleotides 343 to 363) and (reverse) 5′-AGG GAC GTC GTC GTC AGA C-3′.

### Mouse hippocampal slice culture

Hippocampal slice cultures were prepared from P7 rat brains. The 300-μm-thick brain slices were obtained by vibratome sectioning (Leica VT1200, Leica Biosystems) in chilled MEMp [50% (*v*/*v*) minimum essential medium (MEM), 25 mM HEPES, and 2 mM glutamine without antibiotics, adjusted to pH 7.2–7.3 with 1 M NaOH]. The slices were transferred onto semi-porous membrane inserts (Millipore, 0.4 μm pore diameter, Schwalbach, Germany). Intact slices were cultured at 37 °C and 5% CO_2_ in a standard medium MEMi [50% (*v*/*v*) MEM, 25 mM HEPES, 25% (*v*/*v*) HBSS, 25% (*v*/*v*) heat-inactivated horse serum, 2 mM glutamine, 1 ml of penicillin/streptomycin solution, and 0.044% (*v*/*v*) NaHCO_3_, adjusted to pH 7.2–7.3 with 1 M NaOH]. The medium was changed every other day. The slices were cultured for an additional 14 days. Anterograde axonal tracer of biocytin was placed on the entorhinal cortex, dentate gyrus, and CA3 at DIV 15. The hippocampal slices were fixed with 4% PFA at DIV 16. Biocytin was visualized using the ABC-DAB method.

### Cresyl violet staining

Cresyl violet acetate crystal powder was dissolved in distilled water. The solution was then filtered with Whatman paper. Paraffin section slides were immersed in xylene and rehydrated by passing the tissue through decreasing concentrations of ethanol (100 to 70% ethanol). The slides were then immersed in cresyl violet staining solution for 5 min and washed in distilled water. The slices were dehydrated by passing the tissue through a series of ascending alcohol concentrations (70 to 100% ethanol). The final two immersions were in xylene solution. The slides were examined with a microscope (Aperio ScanScope slide scanner), and images were captured with ImageScope software. Indicated box area of pyramidal neurons and number of neurons were measured using ImageJ software.

### Immunofluorescence

The hippocampal slices were fixed in 4% paraformaldehyde for 15 min, permeabilized in PBS containing 0.25% Triton X-100 for 1 h, and blocked in 1% BSA for 1 h. Slices were immunostained using primary antibodies and the appropriate Alexa Fluor 594 goat anti-rabbit and Alexa Fluor 488 goat anti-mouse secondary antibodies. Nuclei were counterstained with DAPI. Immunostained images were acquired using a laser scanning confocal microscope (LSM 710, Carl Zeiss, Germany). Fluorescent images were quantified on a pixel-by-pixel basis using a micro from the Zeiss ZEN software.

### TUNEL assay

Terminal deoxynucleotidyl transferase dUTP nick-end labeling (TUNEL) assay was performed according to the manufacturer’s instructions (cat. G3250, Promega Corporation, Madison, WI, USA) In brief, paraffin-embedded section slides were immersed in xylene and then rehydrated by passing the tissue through graded ethanol (100%, 95%, 75%, and 50%) for 3 min each at room temperature. The slides were treated with reagent mixture (fluorescein-labeled nucleotide mixture and rTdT enzyme and equilibration buffer) and incubated for 1 h at 37 °C.

### Passive avoidance test

The passive avoidance test was performed 4 weeks after IVH induction (at P32) in identical bright and dark compartments, separated by a guillotine door. In the acquisition trial, mice were initially placed in the bright compartment, and the door between the two compartments was opened 10 s later. When mice entered the dark compartment, the door was automatically closed, and an electrical foot shock (0.5 mA) of 10-s durations was delivered through stainless steel rods. When mice were placed in the bright side of a box for step-through passive avoidance, they quickly entered the dark side. Mice were conditioned using an electronic foot shock after entering the dark compartment and hesitated to re-enter the dark compartment when tested 24 h later. In the test, the maximum latency was 180 s.

### Y-maze test

The Y-maze test was performed 4 weeks after IVH induction (at P32) to assess hippocampal-dependent short-term memory function [18]. The Y-maze consists of three horizontal arms separated by 120° angles. After 10 min of adaptation in the maze arms, rat arm alterations were recorded over 10 min. The rest time between acclimatization trial and testing trial was about 2 h. Spontaneous alteration was defined as entry into all three arms consecutively. Between the tests, each arm was thoroughly cleaned. Spontaneous alteration was calculated as follows: % Spontaneous alternation = [(Number of alternations)/(Total entries − 2)] × 100. The number of arm entries served as an indicator of locomotor activity.

### Statistical analysis

The data were expressed as means ± standard error of the mean of three independent experiments with triplicate measurements. In consideration of mortality, more than 14 rat pups were assigned to each group. All studies were performed in a blinded manner. A statistical comparison between the groups was performed by one-way analysis of variance (ANOVA). All of the data were analyzed using SPSS (IBM, Armonk, NY, USA).

## Results

### hUCB-MSC treatment attenuates neuronal loss in the hippocampus after severe IVH injury

**Fig. 1:**
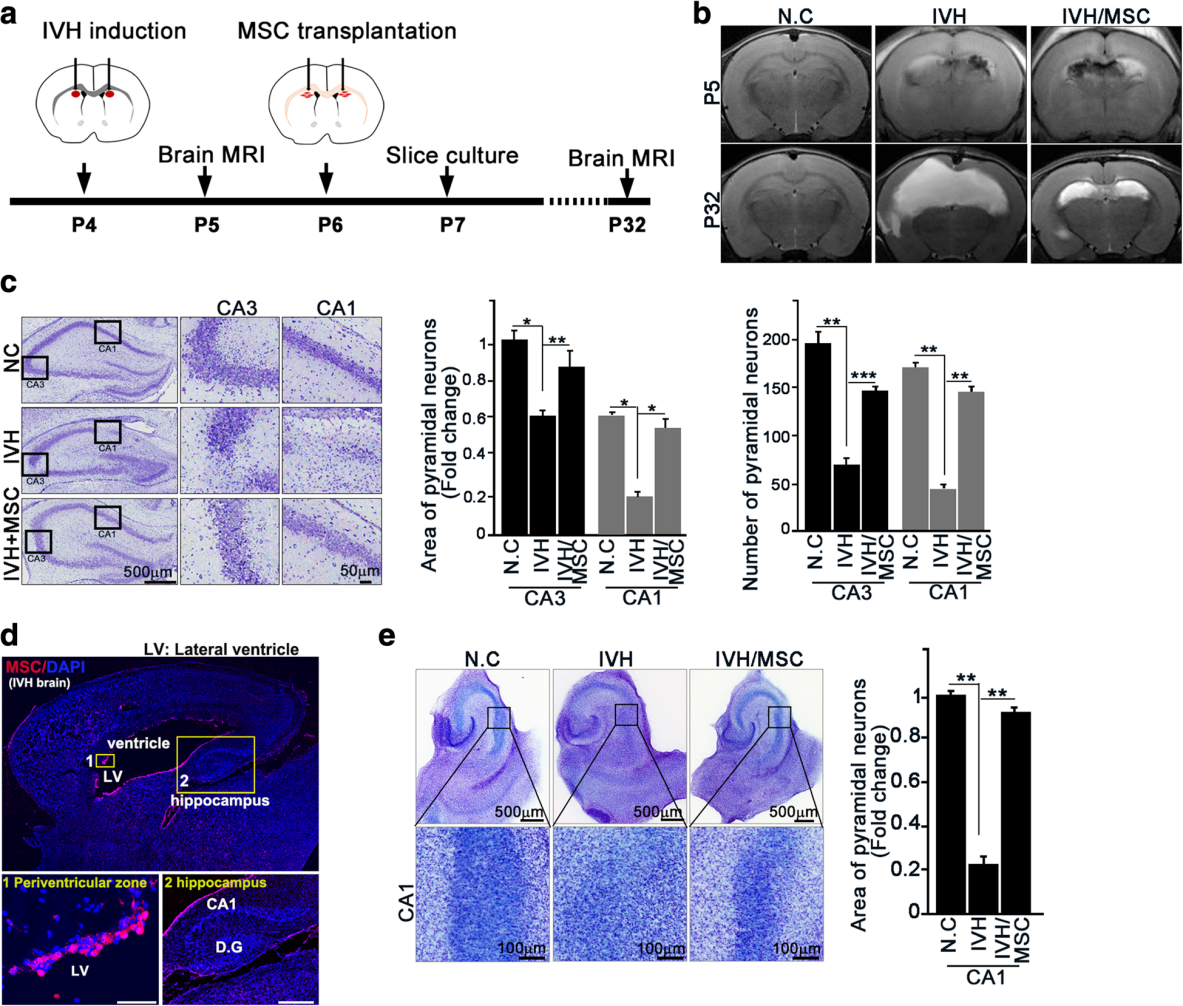
hUCB-MSC treatment prevents neuron loss in the hippocampus after severe IVH injury. **a**, **b** In P4 rat pups, IVH was induced by IC injection of a total of 200 μl of fresh maternal whole blood (100 μl each into the right and left ventricles). Normal control rats received a sham operation without IC blood injection. At P5 (1 day after IVH), severe intraventricular hemorrhage (IVH) was confirmed by brain magnetic resonance imaging (MRI), and IVH rat pups showing minimal or non-visible IVH on the brain MRI were excluded. At P32 (26 days after MSC treatment), IVH damage and its recovery by MSC treatment were measured by MRI. **c** At P7 after animal modeling of intraventricular hemorrhaging, rat brain was embedded in paraffin. Serial hippocampal coronal sections were stained with cresyl violet (left). The area of pyramidal neurons and number of pyramidal neurons were quantified using ImageJ software (right). **d** After severe intraventricular hemorrhage and transplantation of hUCB-MSCs, P7 rat brain was fixed with 4% PFA. Mesenchymal stem cells were stained with anti-human mitochondria antibody. Scale bar, 50 μm (periventricular zone), 500 μm (hippocampus). D.G: dentate gyrus. **e** In P7 rats, entorhinal-hippocampal slice cultures were prepared after intraventricular hemorrhage. Slices are stained with cresyl violet at DIV7 (left). The area of pyramidal neurons was quantified in a black box in ImageJ software (right). MSC markedly protected against neuronal cell death in the CA1 region of the IVH model. Images shown here are representative of at least three independent experiments (*n* ≥ 15 per group). Data are shown as mean ± SEM. *p < 0.05, **p < 0.005 versus the indicated group

hUCB-MSCs were provided by Medipost Co., Ltd., Seoul, Korea [15, 16, 19, 20]. To determine whether treatment with hUCB-MSCs had an effect on neuron loss in the hippocampus of brain-injured newborn rats, we employed a severe IVH injury rat pup model [15–17]. We inflicted severe IVH injury on postnatal day 4 (P4) in rats. At 2 days post-injury, intracerebroventricular administration of MSCs (ICV-MSC) was performed with a stereotaxic frame (Fig. 1a). Successful induction of IVH and recovery from brain injury by MSCs were confirmed by brain magnetic resonance imaging (MRI) after injury (P5) and MSC treatment (P32) (Fig. 1b). To monitor whether IVH-induced brain damage was linked to the neurons in the hippocampus, we collected brain tissue 3 days post-IVH. Obvious neuron loss was observed in the hippocampus, especially in the CA1 regions, of IVH-induced rat brains compared with control rat brains. Treatment with hUCB-MSCs notably decreased neuron loss in the hippocampus (Fig. 1c). To ensure tracking of transplantation of MSCs, we introduced PKH26 (yellow-orange fluorescent dye with long aliphatic tails) pre-labeled MSCs into rat brain after injury, and we were able to track PKH26-positive cells (red) within the periventricular area and hippocampus area of rat brain (Fig. 1d).

To further delineate neuronal damage in the hippocampus, we performed a rat entorhinal-hippocampus (EH) organotypic slice co-culture (OSC) using day 3 post-IVH brains (P7) with or without ICV-MSC; this resulted in a well-preserved cytoarchitecture closely reflecting the corresponding maturation schedule in vivo and is known to be an effective method to study neuronal growth [21, 22]. Consistent with our coronal section of the brain tissue samples, slice cultures collected after 1 day post ICV-MSC (3 days of post-IVH; P7), we observed that IVH elicited impairment of the CA1 regions, and treatment with hUCB-MSCs efficiently negated neuronal loss in the hippocampus (Fig. 1e), suggesting that hUCB-MSC treatment was responsible for recovery from IVH-induced hippocampal injury.

### hUCB-MSC treatment promotes neuronal survival in the hippocampus through activation of BDNF signaling

**Fig. 2:**
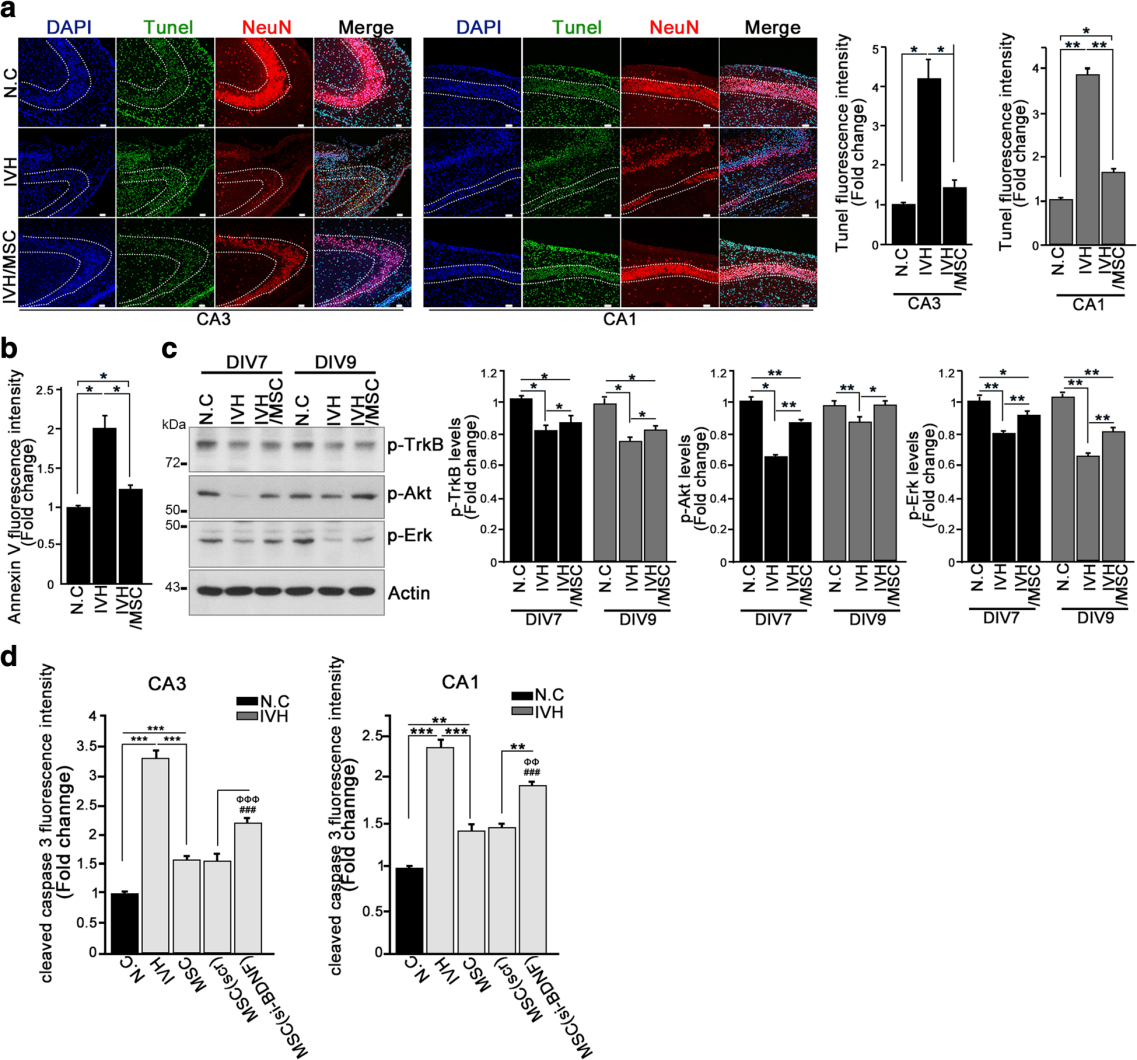
hUCB-MSC treatment promotes neuronal survival in the hippocampus through activation of BDNF signaling. **a** TUNEL staining and immunohistochemistry were performed using sections of paraffin block (P7). DNA was stained with DAPI (blue). TUNEL-positive nuclear staining is green. Neurons were stained with NeuN, a neuronal marker (red). Representative images show the hippocampal CA1 and CA3 regions (left). Scale bar, 50 μm. Quantification of relative immunofluorescence intensity of TUNEL signal is shown as a bar graph (right). Data are shown as mean ± SEM; *p < 0.05 and ** *p* < 0.005 versus the indicated group (*n* ≥ 15 per group). **b** Rat brain paraffin sections were stained with annexin V and NeuN. Bar graph shows the annexin V fluorescence intensity. A representative figure is shown in Additional file 1: Figure S1A. Images shown here are representative of at least three independent experiments. Data are shown as mean ± SEM. *p < 0.05 versus the indicated group. **c** Whole rat brains were dissected and lysed at the indicated time points and applied for immunoblotting analysis with the indicated antibodies. Bar graph shows quantified amounts of p-TrkB, pAKT, and p-Erk. Data are representative of at least three independent experiments. Data are shown as mean ± SEM; *p < 0.05 and **p < 0.005 versus the indicated group. **d** Paraffin sections were stained with cleaved caspase3 and NeuN. Bar graph shows the cleaved caspase 3 fluorescence intensity. Data are shown as mean ± SEM from three independent experiments, and a representative image is shown in Additional file 1: Figure S1D. *n* ≥ 15 per group. **p < 0.005 and ***p < 0.0005 versus the indicated group. ^###^*p* < 0.0005 versus N.C, ^ΦΦΦ^*p* < 0.0005 versus IVH

As we found that IVH-induced brain injury was responsible for apparent neuronal loss and that hUCB-MSC treatment after IVH injury conserved the normal architecture of the hippocampus, we wondered whether loss of neurons could be due to neuronal apoptosis. To determine neuronal apoptosis, we generated paraffin blocks of three groups of rats (negative control, IVH injury only, and hUCB-MSC treatment after IVH injury) and employed double staining with NeuN, a neuron marker, and terminal deoxynucleotidyl transferase dUTP-biotin nick end labeling (TUNEL), an apoptotic marker, or annexin V. By 3 days post-IVH without treatment with hUCB-MSCs, the dissected hippocampal CA3-CA1 regions showed notable TUNEL staining, whereas the hippocampi from hUCB-MSC-treated rat brains after IVH injury showed only around one quarter of the TUNEL fluorescence intensity of the IVH injury model (Fig. 2a). Moreover, annexin V applied onto the paraffin sections of IVH injury with or without hUCB-MSC treatment demonstrated an obvious decrease in signal in the hUCB-MSC treatment group (Fig. 2b; Additional file 1: Figure S1A). Thus, our data indicated that IVH injury probably resulted in neuronal apoptosis, and hUCB-MSC treatment decreased neuronal death, thereby enhancing neuronal survival in the hippocampus.

To identify the potential mechanisms of action of hUCB-MSCs in the hippocampus, we tested BDNF signaling. We collected brain tissues from rat hippocampus after injury and evaluated TrkB receptor activation, a well-known selective receptor for BDNF, as well as Akt and extracellular signal-related kinase (Erk)/mitogen-activated protein kinase (MAPK) activation. These are representative downstream signaling molecules of BDNF signaling. In the control group, TrkB receptor was constantly activated by tyrosine phosphorylation (Fig. 2c, first panel, first and fourth lanes). However, under IVH injury conditions, TrkB activation was significantly decreased 3 days post-injury (P7) and remained low until 5 days post-injury (P9) (Fig. 2c, first panel, second and fifth lanes). Of importance, in the group of rats receiving hUCB-MSC treatment after IVH injury, TrkB activation was highly conserved and gradually increased over the study compared to the IVH injury only group (Fig. 2c, first panel, third and sixth lanes). In addition, Akt and Erk activation, represented by their phosphorylation, was similar to TrkB activation (Fig. 2c, second and third panels), suggesting that treatment with hUCB-MSCs upregulated intracellular BDNF/TrkB signaling.

To elucidate whether the perturbation against neuronal apoptosis by hUCB-MSC treatment was indeed due to BDNF signaling, we conducted an immunohistochemistry study utilizing BDNF-depleted hUCB-MSCs that were transfected with BDNF-Si-RNA for knockdown of BDNF or scrambled RNA transfected into control hUCB-MSCs-injected rats after IVH induction. This experiment was also performed in three groups of rats (negative control, IVH injury only, hUCB-MSC treatment after IVH injury) and those receiving hUCB-MSC (scramble siRNA control) treatment after IVH injury. BDNF depletion in hUCB-MSCs was evaluated by microscopy and immunoblotting (Additional file 1: Figure S1B and C). Relative to control hUCB-MSC injection, the immunoreactivity of cleaved caspase-3 was highly elevated in the CA1 and CA3 regions of BDNF-lacking hUCB-MSC injection groups, indicating that BDNF is a potent neuroprotective molecule in hUCB-MSCs (Fig. 2d; Additional file 1: Figure S1D). Therefore, we speculated that IVH injury-induced neuron loss and apoptosis might be related to the destruction of BDNF signaling.

### hUCB-MSC treatment enhanced neurogenesis in the hippocampus after neuron loss

**Fig. 3:**
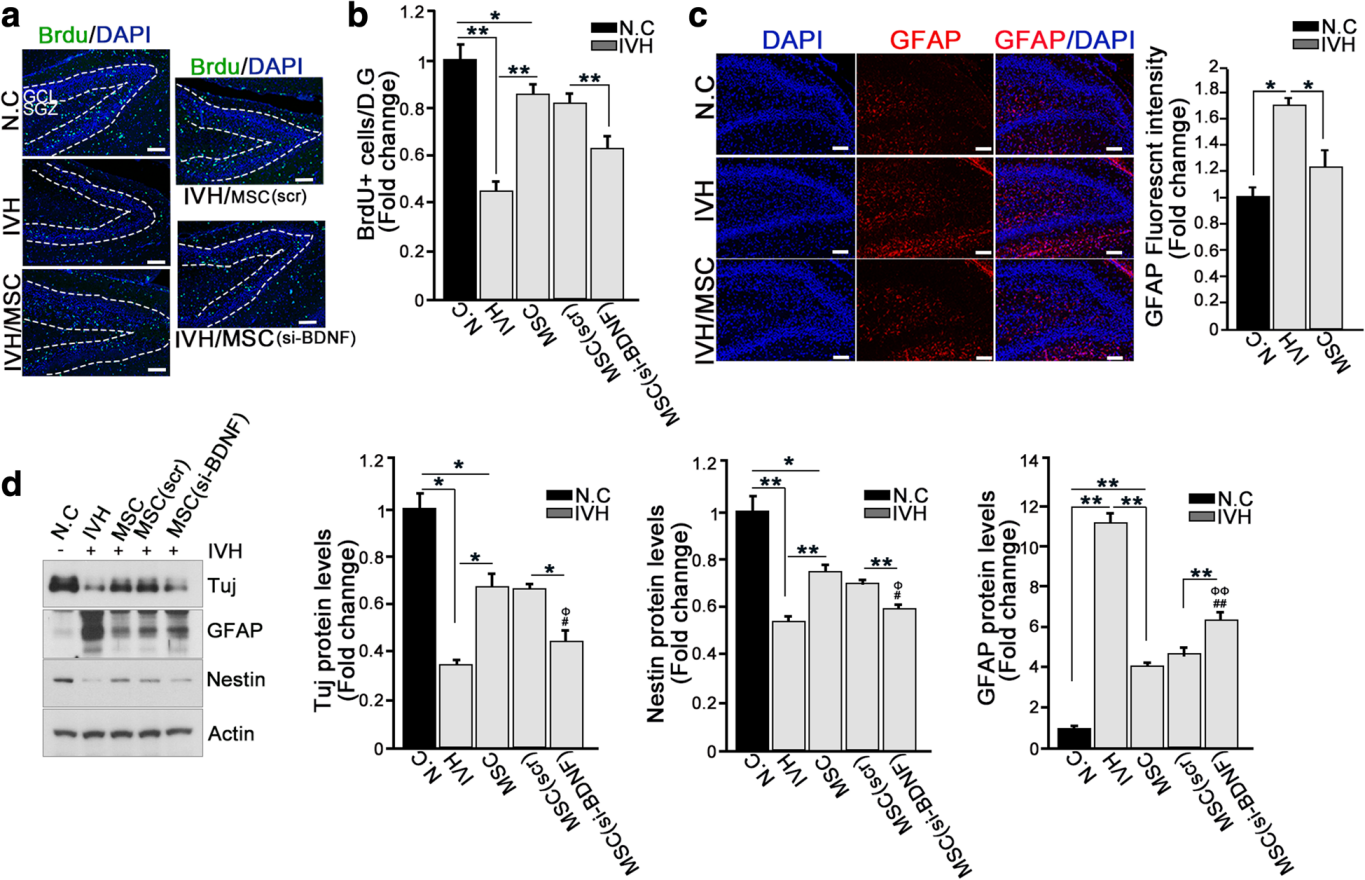
hUCB-MSC treatment enhanced neurogenesis in the hippocampus after neuron loss. **a**, **b** Effect of MSCs on neurogenesis in the hippocampal dentate gyrus following IVH. At 3 days after severe intraventricular hemorrhage, rat was injected intraperitoneally with 5-bromo-deoxyuridine (BrdU, 100 mg/kg). At 24 h after BrdU i.p. injection, the rat brain was fixed with 4% PFA for 48 h. To determine neurogenesis, a paraffin section was stained with anti-BrdU antibody (green) and DAPI (blue). Scale bar, 100 μm. Data are shown as mean ± SEM; **p < 0.005 versus the indicated group. **b** Quantification of BrdU+ signal is shown as a bar graph. Images shown here are representative of at least three independent experiments, and each value represents the mean ± SEM of triplicate measurements. *p < 0.05 and **p < 0.005 versus the indicated group. **c** After severe IVH and transplantation of hUCB-MSCs, P7 rat brain was fixed with 4% PFA for 48 h. Glial cells were stained with anti-GFAP antibody (red). Nuclei were counterstained with DAPI (blue). Scale bar, 100 μm. The bar graph shows GFAP fluorescence intensity. Data are shown as mean ± SEM; *n* = 3. *p < 0.05 versus the indicated group (right). **d** Rat hippocampi of IVH/MSC rat models were dissected at p7. Lysates were subjected to IB with the indicated antibodies. Data are representative of three independent experiments. Data are shown as mean ± SEM; *n* ≥ 15 per group. *p < 0.05 and **p < 0.005 versus the indicated group. ^##^*p* < 0.005 versus N.C, ^ΦΦ^*p* < 0.005 versus IVH

To maintain the normal functional architecture of the hippocampus, it is necessary to either prevent neuron loss or increase neurogenesis. We hypothesized that, in addition to protection against neuronal apoptosis, hUCB-MSC treatment would increase neurons in the hippocampi of newborn rats. To test this hypothesis, five groups of rats (negative control, IVH injury only, hUCB-MSC treatment after IVH injury, hUCB-MSC (scramble siRNA control) treatment after IVH injury, and hUCB-MSC (siRNA for BDNF) treatment after IVH injury) were injected intraperitoneally with the proliferation marker 5-bromo-deoxyuridine (BrdU) 24 h prior to the tissue collection, and BrdU-positive cells were quantified in the dentate gyrus (DG) where neurogenesis primarily occurs in the hippocampus. Consistent with our finding that IVH injury resulted in notable neuron loss, the numbers of BrdU-positive cells were greatly reduced. Treatment with hUCB-MSCs notably attenuated these deficits (Fig. 3a). To further clarify the neurogenic effects of hUCB-MSCs after IVH injury, we employed a hippocampal slice culture system and maintained another 7-day hippocampal slice prior to BrdU treatment. Importantly, BrdU-positive cells migrating into the granular cell layer (GCL) from the subgranular zone (SGZ) were increased in the dentate gyrus, indicating the neurogenic effects of hUCB-MSC treatment. Migration of newborn neurons into the GCL from the SGX is an important step in hippocampal neurogenesis and serves as a strong indicator of neurogenic activity [23, 24]. However, injection of BDNF-lacking hUCB-MSCs failed to induce neurogenesis, resulting in a much lower number of BrdU-positive cells in the DG compared to control hUCB-MSC-treated tissue (Fig. 3a; Additional file 2: Figure S2A). This result suggests that the neurogenic function of hUCB-MSCs is, at least in part, derived from BDNF.

One of the most commonly used criteria for neural progenitor cells is the expression of the intermediate filament nestin. In our hippocampal slice culture model of injured rats, nestin-expressing neurons in the DG were diminished in the IVH injury group but noticeably elevated in the hUCB-MSC treatment group. Likewise, we demonstrated that the expression of neuron-specific class III beta-tubulin (Tuj1) was positively correlated with nestin expression (Additional file 2: Figure S2B). However, astrocyte-specific marker glial fibrillary acidic protein (GFAP) expression was notably observed in the IVH injury group, while it was decreased after hUCB-MSC treatment (Fig. 3c). Reactive astrogliosis is known to impair neurogenesis and was inversely correlated with nestin expression (Fig. 3c), suggesting that nestin-expressing cells in the DG were distinguishable from reactive astrocytes and thus were representative of neuronal progenitors. In accordance with our immunohistochemistry analysis, nestin and Tuj1 expression levels were upregulated, while GFAP expression level was reduced in in the hUCB-MSC treatment group compared with the IVH injury group (Fig. 3d). The neurogenic function of hUCB-MSCs was greatly curtailed in the DG of BDNF-lacking hUCB-MSC groups, which showed much lower nestin expression compared with control hUCB-MSC-injected tissues (Fig. 3d). Taken together, our data suggest that treatment with hUCB-MSCs promoted neurogenesis in the DG of the hippocampus, most likely through BDNF signaling, to compensate for neuron loss.

### The BDNF-Akt/Erk-CREB axis contributes to neurogenesis in the hippocampus after IVH injury

**Fig. 4:**
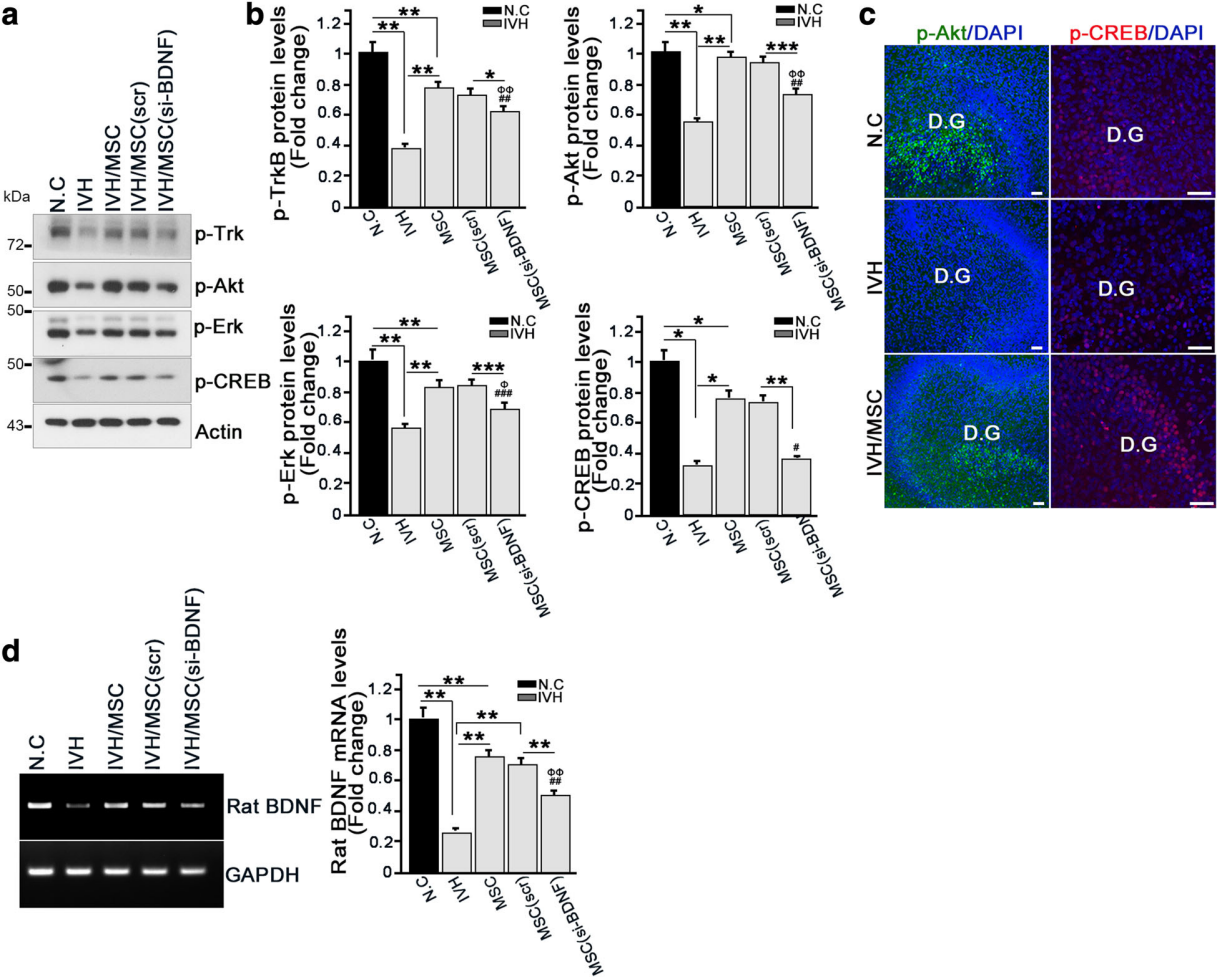
The BDNF-Akt/Erk-CREB axis contributes to neurogenesis in the hippocampus after IVH injury. **a**, **b** Rat hippocampus were dissected and lysed at p7 in IVH/MSC rat models. Lysates were subjected to IB with the indicated antibodies (left). Amounts of p-TrkB, p-Akt, p-Erk, and p-CREB protein were determined by IB (right). Images shown here are representative of at least three independent experiments, and each value represents the mean ± SEM of triplicate measurements; *p < 0.05 and ** *p* < 0.005 versus indicated groups, ^##^*p* < 0.005 versus N.C, ^###^*p* < 0.0005 versus N.C, ^Φ^*p* < 0.05 versus IVH, ^ΦΦ^*p* < 0.005 versus IVH. **c** Hippocampal slice cultures were prepared after IVH. Slices were cultured for an additional 7 days and fixed with 4% paraformaldehyde. Slice was stained with p-ATK and p-CREB antibody (green). Nuclei were counterstained with DAPI stain (blue). Scale bar, 50 μm. **d** Rat hippocampus was dissected at P7. RNA was isolated from the hippocampus, and the mRNA levels of rat BDNF were determined using species-specific primers (left). Quantification of rat BDNF mRNA measurements from three independent experiments is shown on the right. Data are shown as mean ± SEM; **p < 0.005 versus the indicated group. ^##^*p* < 0.005 versus N.C, ^ΦΦ^*p* < 0.005 versus IVH

BDNF signaling occurs through cAMP-response element binding protein (CREB) activation, a transcription factor that functions in neurogenesis and memory and binds to cAMP-response element sites in the BDNF promoter to enhance gene transcription [25–27]. Moreover, the important downstream mediators of BDNF-TrkB activation are necessary to induce neuronal activity-dependent CREB phosphorylation, namely, Akt activates CREB through its downstream effector, GSK3 beta phosphorylation, and ERK/MAPK signaling [28, 29]. Thus, we examined whether hUCB-MSC-mediated neurogenesis was involved in BDNF/TrkB-mediated CREB transduction. Compared with the control group, under IVH injury conditions, the levels of phosphor-TrkB-, phosphor-Akt-, and phosphor-Erk as well as those of phosphor-CREB decreased and were barely detectable, whereas under hUCB-MSC treatment conditions, TrkB-Akt/Erk-CREB activation was dramatically increased in the isolated hippocampus extract. However, BDNF-lacking hUCB-MSCs could not restore activation of intracellular signaling in the TrkB-Akt/Erk-CREB axis (Fig. 4a, b).

In the hippocampal slice culture model, the activation of Akt and CREB signaling was prominently detected in the DG, where neurogenesis notably occurred under hUCB-MSC treatment conditions, while both Akt and CREB activation was robustly diminished in the DG of IVH injury rats (Fig. 4c). These data imply that neurogenesis in the DG after hUCB-MSCs treatment could be affected through the activation of CREB signaling in the hippocampus.

To investigate whether the increased CREB phosphorylation after TrkB activation by BDNF secreted from hUCB-MSCs was able to enhance rat BDNF transcription, we determined the mRNA levels of BDNF using species-specific primers in the hippocampus. Indeed, concurrent with CREB activation, rat BDNF mRNA in hUCB-MSC-treated hippocampus was increased. In contrast, mRNA of rat BDNF was more weakly detected in the IVH injury group without hUCB-MSC treatment (Fig. 4d). Regardless of hUCB-MSCs treatment, we could not detect human BDNF mRNA (data not shown). BDNF-lacking hUCB-MSC treatment failed to increase BDNF transcription levels (Fig. 4d). Because of the limitations of antibody specificity due to the high homology between rat and human BDNF proteins, we were unable to clearly determine whether it was the human BDNF secreted from hUCB-MSCs binding to rat TrkB receptors to initiate intracellular TrkB-CREB signaling. However, based on our results showing that BDNF-lacking hUCB-MSCs could not invoke TrkB-CREB signaling, presumably because of the high amino acid homology between human and rat BDNF, it is likely that it was the human BDNF binding to the rat TrkB receptors and turning on intracellular signaling leading to CREB activation, which subsequently enhanced CREB-mediated rat BDNF transcription, thereby upregulating rat BDNF secretion.

### hUCB-MSC treatment contributes to the recovery of IVH injury-mediated lesions in the hippocampal trisynaptic circuit

**Fig. 5:**
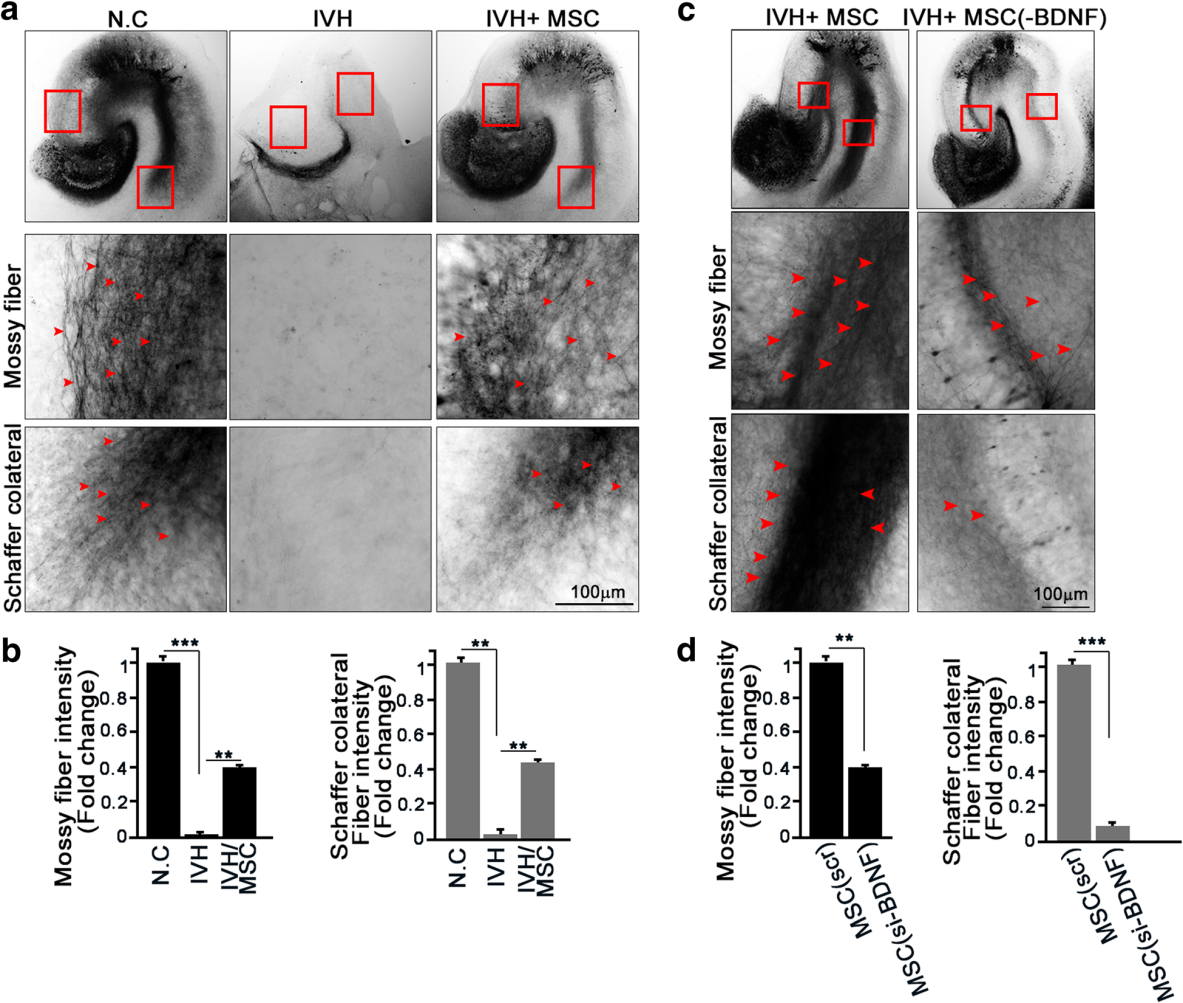
hUCB-MSC treatment contributes to the recovery of IVH injury-mediated lesions in the hippocampal trisynaptic circuit. **a**, **b** Hippocampal slices of IVH/MSC rat models were cultured at P7. The anterograde axonal tracer biocytin was placed on the entorhinal cortex, D.G, and CA3 at DIV8 and fixed with 4% PFA. Biocytin was visualized using the ABC-DAB method. Red arrows indicate mossy fibers (upper) and Schaffer collateral fibers (bottom). Image shown here is representative of at least three independent experiments. Scale bar, 100 μm. **b** Bar graph shows mossy fiber intensity and Schaffer collateral fiber intensity. Data are shown as mean ± SEM; *n* ≥ 20 per group. **p < 0.005 and ***p < 0.0005 versus the indicated group. **c** To determine the effect of MSCs (BDNF) on hippocampal trisynaptic fiber, MSCs (scr) or MSCs (si-BDNF) were transplanted at p6 following IVH. After 7 days of slice culture, the anterograde axonal tracer biocytin was placed on the slice. Red arrows indicate the mossy (middle) and Schaffer collateral (bottom) fibers. Scale bar, 100 μm. **d** Bar graph shows mossy fiber intensity and Schaffer collateral fiber intensity. Image shown here is representative of at least three independent experiments. Data are shown as mean ± SEM; **p < 0.005 and ***p < 0.0005 versus the indicated group

Within the hippocampus, the information passes through a trisynaptic circuit (DG➔CA3➔CA1). These connections are depicted in Additional file 3: Figure S3A. We found that IVH injury led to defects in the hippocampus and that hUCB-MSC treatment prevented hippocampal destruction and induced neurogenesis. We determined whether IVH injury affected a particular synaptic path and whether hUCB-MSCs injection could overcome this to regenerate that synaptic path. Despite the post-injury tissue with no exposure to hUCB-MSCs, evidence of axon projection through the perforant path was detected, and treatment with hUCB-MSCs increased the numbers of axons (Additional file 3: Figure S3B). However, using biocytin labeling, we showed that IVH injury robustly destroyed the CA3 and CA1 routes, revealing a nearly invisible neural path through both the mossy fiber and Schaffer collateral paths (Fig. 5a, b). Importantly, hUCB-MSC injection rescued the neural network of the mossy fiber and Schaffer collateral paths. These data imply that hUCB-MSC injection after IVH injury prevents destruction of the hippocampal neural network.

Next, we determined whether the neural repair function of hUCB-MSCs was derived from BDNF effects. The administration of hUCB-MSCs significantly conserved the neural path and induced neurite outgrowth, whereas treatment with BDNF-lacking hUCB-MSCs failed to shield the devastation of either the mossy fiber or Schaffer collateral paths, which showed relatively less neurite regeneration (Fig. 5c, d). Immunoblotting analysis with the above hippocampal extract supported the hypothesis that impairment of BDNF signaling in hUCB-MSCs attenuated their protective function, which was elucidated by the decline of phosphor-Akt and phosphor-CREB levels in the hippocampus of BDNF-lacking hUCB-MSC-injected rats (Additional file 3: Figure S3C). Moreover, the neural marker Tuj1 was significantly downregulated, whereas the astrocyte marker GFAP was upregulated in in the hippocampi of BDNF-lacking hUCB-MSC-injected rats compared to those of the control hUCB-MSC-injected group (Additional file 3: Figure S3C). Taken together, these results indicate that hUCB-MSC treatment triggered the neural repair process through the BDNF-Akt-CREB axis in the hippocampus and possibly through protective synaptic functions.

### hUCB-MSC treatment reinstates behavioral function through BDNF signaling

**Fig. 6:**
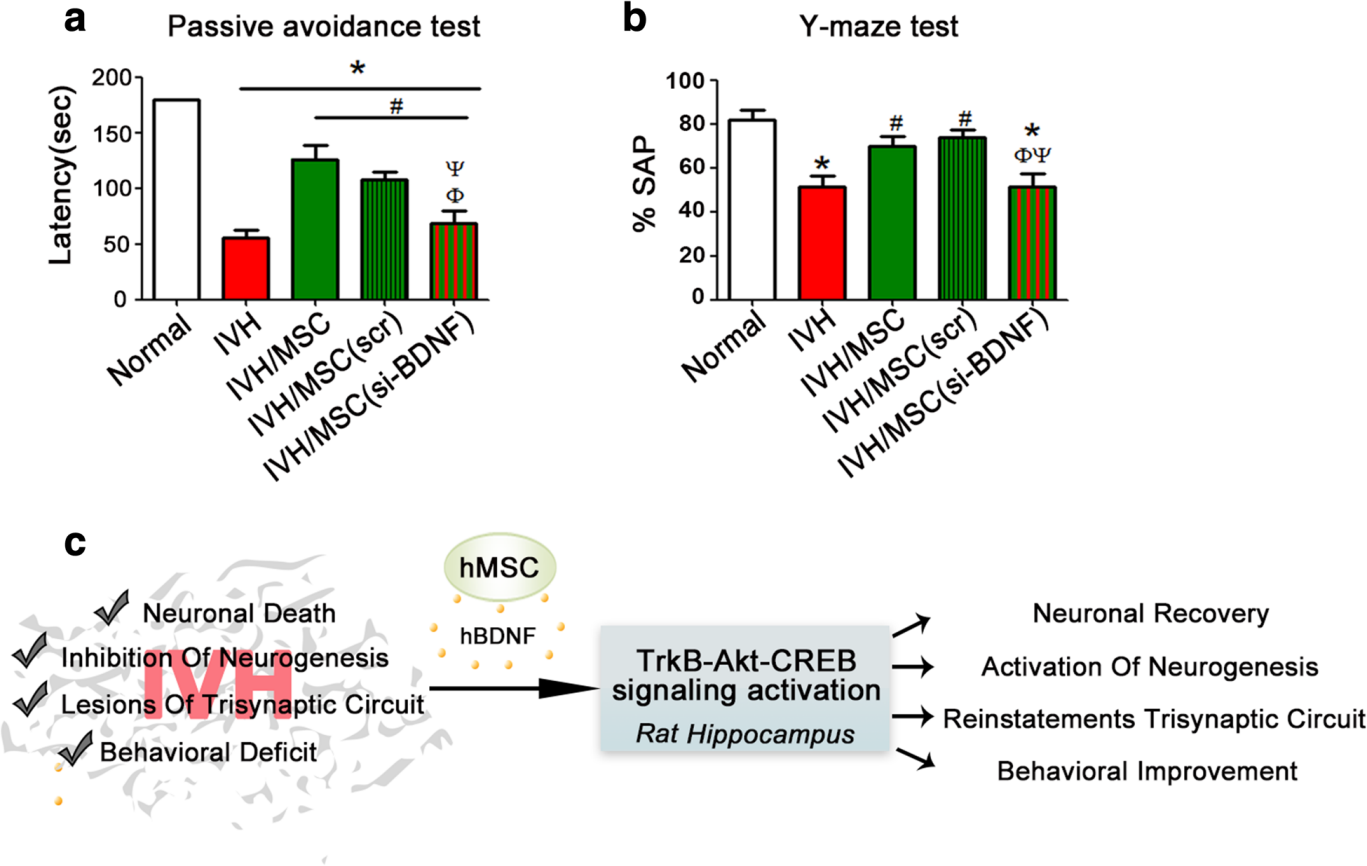
hUCB-MSC treatment reinstates synaptic function through BDNF signaling. Learning and memory functional outcomes on passive avoidance test (**a**) and Y-maze (**b**) at P32. Data are expressed as mean ± standard error of the mean (SEM). *p < 0.05 versus normal, ^#^*p* < 0.05 versus IVH control, ^Φ^*p* < 0.05 versus IVH+MSC, ^Ψ^*p* < 0.05 versus IVH+scrambled siRNA-transfected MSCs. **c** Schematic diagram of hUCB-MSC-induced BDNF signaling after IVH in rat hippocampus

To ascertain if hUCB-MSC treatment-mediated reconstruction of the neural path was actually endowed with synaptic function, we monitored behavioral recovery, focusing on passive avoidance and Y-maze testing; these are classically used to assess neurocognition that requires hippocampal function. In passive avoidance tasks, the time to enter the dark compartment in the post-IVH injury group (a positive group) was markedly decreased compared to that of the non-injury control rats (*p* < 0.001) (Fig. 6a). Importantly, the hUCB-MSC treatment groups notably recovered movement behavior compared to the IVH injury group, which reflected normal exploratory behavior in an aversive environment. Moreover, spontaneous alterations in the Y-maze were significantly lower in the post-IVH injury group compared with the non-injury control group (*p* < 0.001), while the hUCB-MSC-treated group showed remarkably increased alterations indicating functional recovery promoted by the hUCB-MSC injection (*p* < 0.001). Furthermore, while the hUCB-MSC-treated group displayed significant improvement in latency and spontaneous alteration as compared to the IVH injury group, BDNF-lacking hUCB-MSC-injected rats exhibited high latency scores in the passive avoidance test and low alteration scores in the Y-maze test, implying failure of functional reconstitution (Fig. 6a, b). Collectively, our behavioral test results demonstrated that hUCB-MSC injection was able to provide a considerable improvement of synaptic function after severe IVH.

## Discussion

Recent preclinical studies have shown the therapeutic potential of MSC treatment for brain diseases mainly involved in cognitive functions due to neuron loss including neurodegenerative disease and traumatic brain injury (TBI) [30–32]. Similarly, our studies suggested that treatment with hUCB-MSCs in infants with IVH might be a reasonable therapeutic strategy [33]. In the current study, we demonstrated that neural cells in the hippocampus, including the dentate gyrus, were highly vulnerable to IVH-induced brain injury, and their loss caused neurocognitive dysfunction. This provides evidence that hUCB-MSC treatment attenuates neuron loss due to IVH injury-induced neuronal death and enhances neurogenesis, thereby contributing to the recovery of synaptic pathways in the hippocampus through BDNF-TrkB-CREB signaling. Moreover, our behavioral tests demonstrated that treatment with hUCB-MSCs after IVH improved learning and memory deficits, reflecting the recovery of behavioral function in the hippocampus. Thus, our data potentially explain the benefits of treatment with hUCB-MSCs after neuron loss and highlight the role of BDNF-CREB signaling as a key element in recovery from brain damage.

Although direct injection of BMSCs into the lateral ventricle in a rat depression model showed migration of BMSCs to the dentate gyrus and CA1 and CA3 regions of the hippocampus and increased neurogenesis [34], there has been no direct evidence of changes in neurogenesis following hUCB-MSC treatment. Here, we demonstrated that hippocampal neurons are vulnerable to IVH-induced brain injury and that hUCB-MSCs are able to prevent neuron loss in the hippocampus while also enhancing their proliferation in the dentate gyrus (Figs. 2 and 3). Moreover, synaptic circuits such as the mossy fiber and Schaffer collateral paths are affected by either neuron loss or the structure of the neural circuit as detected with the anterograde axonal tracer biocytin after IVH injury in an organotypic slice culture model, and hUCB-MSC treatment apparently restored the impaired synaptic path (Fig. 5).

How do hUCB-MSCs exert their functional benefits regarding neuronal loss and diminution of neurogenesis effects? In addition to trans-differentiation or cell fusion, one of the major mechanisms MSCs use to promote neural functional recovery is secretion of neurotrophic factors [35, 36]. Canonical BDNF-TrkB receptor signal transduction is important in intracellular signaling for neuronal survival, neurogenesis, and synaptic plasticity and plays a crucial role in learning and memory functions in the central nervous system [37–39]. Phosphorylated CREB binds to the BDNF promoter and enhances its transcription and is a critical point of convergence in the signaling pathway regulating neuronal survival and neurogenesis, as well as neuronal plasticity [40, 41]. Our data demonstrated that hUCB-MSC treatment provoked TrkB signaling, activating either Akt or Erk, thereby leading to CREB phosphorylation (Figs. 2c and 4), whereas treatment with siBDNF-hUCB-MSCs that lacked BDNF in the siRNA treatment group showed much weaker CREB activation. Compared with the negative control group that was not exposed to IVH injury, the injured hUCB-MSC transplant group showed more than 80% recovery for BDNF-TrkB signaling. However, hUCB-MSC transplantation obviously restored the activation of TrkB-Akt/Erk signaling compared with the IVH injury group. Considering the future need for studies on the optimal source, timing, route, and dose for future clinical introduction of MSC transplantation as a treatment for severe IVH, it may be possible to improve the efficacy of MSC transplantation if the therapeutic time window is determined. We introduced MSCs 2 days after severe IVH injury and/or along the optimal route through intraventricular administration and so cannot rule out the possibility of additional injury to normal brain/tissues. Of interest, in our previous study, we demonstrated that human BDNF released from injected hUCB-MSCs was detected in high levels 1 day after transplantation (P7) but was not detected at 5 days (P11) after transplantation, while the levels of rat BDNF were strikingly high on P7 and were still augmented on P11 [17]. Since we noticed that CREB is activated on 1 day after transplantation (P7) and activated CREB-accelerated transcription of rat BDNF but not human BDNF (Fig. 4d), it is reasonable to assume that transplanted hUCB-MSCs secrete human BDNF that binds to TrkB receptors in rat brain cells due to the high amino acid sequence homology (more than 90%) between human and rat BDNF. This BDNF provoked intracellular signaling such as Akt or Erk activation, which was able to contribute to neuroprotection and neurogenesis, leading to CREB activation. The activated CREB subsequently promoted mRNA expression of rat BDNF, thereby enhancing rat BDNF release. Furthermore, our RT-PCR analysis revealed that only rat BDNF mRNA was increased; we found no detectable increase in mRNA of human BDNF. These data might reflect why we were able to identify relatively high levels of rat BDNF after the human BDNF was no longer measurable. Hence, our study illustrates that in the injured condition that the endogenous rat BDNF production and intracellular signaling were severely impaired due to IVH induced injury, human BDNF secreted from transplanted hUCB-MSCs could be recognized by TrkB receptor in the rat brain cells and reactivating intracellular Akt/Erk-CREB signaling thereby consequently restores the expression of rat BDNF, acting as an extracellular cue to initiate intracellular signaling of host cells (Fig. 6c).

Our findings that hUCB-MSCs after IVH can elicit prominent neurogenesis and recovery of the hippocampal neural circuit could have relevance for performance improvement in behavioral tasks related to hippocampus neurogenesis [42]. Both the passive avoidance and Y-maze tasks are well-documented tests of learning and memory as well as cognition and require hippocampal neurogenesis for synaptic function. The level of active or phosphorylated CREB in the hippocampus is directly related to cognitive decline, and impairment of CREB activity leads to deficits in working memory [43, 44]. In agreement with previous studies, our data clearly showed that hUCB-MSCs promoted improved cognitive recovery and were positively related to BDNF, as lack of BDNF in hUCB-MSCs that possessed much lower levels of phosphorylated CREB in the hippocampus did not improve memory deficits (Fig. 6a, b). Thus, our study suggests a possible molecular mechanism for the therapeutic potential of hUCB-MSCs in neuron loss and neurocognitive dysfunction in IVH and possibly other neurodegenerative diseases characterized by these deficits.

IVH initiates in the germinal matrix, where neuronal-glial precursor cells are enriched in the developing brain and are attributed to the fragility of germinal matrix vasculature that exhibits a paucity of pericytes, immaturity of basal lamina, and lack of GFAP in the astrocyte endfeet. Thus upregulation of GFAP by growth factor such as VEGF that activates a rapid angiogenesis could contribute to IVH protection [2]. Capillary remodeling in the germinal matrix vasculature upon increased VEGF is ascribed to hypoxia distinctive of this brain region perhaps due to high metabolic activity and oxygen consumption of neural progenitor cells. Previous study suggested VEGF and hepatocyte growth factor are ascribed to protective growth factors which are secreted from transplanted hUCB-MSCs under hyperoxic neonatal lung injury [45]. Considering multiple paracrine factors that secreted from MSCs and characteristics of IVH, we cannot rule out the possibility that other cell types such as astrocyte in the brain besides neurons in the damaged region or outside of injured region may contribute to restore angiogenic vessels achieving VEGF from transplanted hUCB-MSCs. Likewise, these secreted growth factors and/or cytokines combine to stimulate neural cells for survival and neurogenesis [46]. It is also possible that in addition to BDNF signaling, hUCB-MSCs treatment may elicit another intracellular signaling such as Wnt/beta-catenin signaling that is known to be involved in the regulation of adult hippocampal neurogenesis [47, 48]. Therefore, BDNF may not completely recapitulate the effects of hUCB-MSCs, and it may not be the only secreted factor that contributes to neuronal survival and neurogenesis in the hippocampus. However, hUCB-MSCs treatment in severe IVH models significantly upregulated BDNF expression instead of other growth factors/cytokines while same hUCB-MSCs treatment in hyperoxic neonatal lung injury models highly upregulated VEGF secretion [49]. Moreover, BDNF signaling is required for not only embryonic but also adult neurogenesis [50], and our data showed that treatment of hUCB-MSCs enhances endogenous TrkB-Akt-CREB signaling as well as the reinstatement of behavioral function. Thus, our study (Fig. 6c) provides causal relationships between behavioral tasks and hUCB-MSC-induced secreted BDNF signaling that might initiate intracellular signaling in the hippocampus, subsequently activating Akt and Erk signaling to result in CREB activation, which, in turn, is responsible for elevating BDNF expression and contributing to the recovery of cognitive function in IVH.

## Conclusions

Our current studies suggested that hUCB-MSC treatment attenuates neuronal loss and enhanced neurogenesis in the hippocampus, stimulating intracellular BDNF-TrkB-Akt/Erk-CREB signaling after severe IVH injury, thereby contributing to the recovery of IVH injury-mediated lesions in the hippocampal trisynaptic circuit along with the behavioral improvements. Therefore, hUCB-MSCs treatment should provide a valuable promising alternative for cell-based therapies in IVH injury.

## Additional files

Additional file 1: **Figure S1.** hUCB-MSC treatment promotes neuronal survival in the hippocampus through activation of BDNF signaling. (a) Paraffin rat brain sections were stained with annexin V (red) and NeuN (green). Nuclei were counterstained with DAPI stain (blue). Images shown here are representative of at least three independent experiments. Scale bar, 50 μm (b and c) MSCs were transfected with siRNA oligonucleotides using Oligofectamine (Invitrogen, Carlsbad, CA, USA) according to the manufacturer’s instructions. From the MSCs, BDNF expression was successfully knocked-down and sustained for at least 48 h after transfection of BDNF siRNA. (d) Neuronal cell death was assessed on the paraffin section at P7. Brain section was stained with cleaved caspase 3 (red) and NeuN (green). Nuclei were counterstained with DAPI stain (blue). Images shown here are representative of at least three independent experiments. Scale bar, 50 μm. (TIF 8489 kb)

Additional file 2: **Figure S2.** hUCB-MSC treatment enhanced neurogenesis in the hippocampus after neuron loss. (a) Hippocampal slice culture was treated with BrdU (10μg/ml) at DIV7. The slice was fixed with 4% PFA at DIV9 and stained with anti-BrdU antibody (green). Nuclei were counterstained with DAPI stain (blue). Scale bar, 50 μm. (b) After severe IVH and transplantation of hUCB-MSCs, P7 rat models were analyzed with an entorhinal-hippocampus organotypic slice co-culture. Slice culture was fixed and stained with the neural stem cell marker nestin (green) and the neuron marker TUJ1 (green) at DIV7. Nuclei were counterstained with DAPI stain (blue). Images shown here are representative of at least three independent experiments. Scale bar, 50 μm. (TIF 3765 kb)

Additional file 3: **Figure S3.** hUCB-MSC treatment contributes to the recovery of IVH injury-mediated lesions in the hippocampal trisynaptic circuit. (a) Schematic diagram of hippocampal trisynaptic circuit. The perforant path is the connectional route from the entortinal cortex to the dentate gyrus. Signal information from the dentate gyrus projects along the mossy fiber to the cornu ammonis area 3 (CA3). Axons from CA3 project to area CA1 pyramidal neurons via Schaffer collateral fibers. (b) The hippocampal slices of IVH/MSC rat models were cultured at P7. The anterograde axonal tracer biocytin was placed on the entorhinal cortex at DIV8 and fixed with 4% PFA. Biocytin was visualized using the ABC-DAB method. Red arrows indicate the perforant fibers. The square boxed area is enlarged in the bottom panel. Images shown here are representative of at least three independent experiments. Scale bar, 100 μm. (c) Rat hippocampi were dissected and lysed at P7 in IVH/MSCs rat models. Lysates were subjected to IB with the indicated antibodies. Images shown here are representative of at least three independent experiments. (TIF 3251 kb)

## Abbreviations

BDNF: Brain-derived neurotrophic factor
DG: Dentate gyrus
ERK: Extracellular signal-related kinase
GCL: Granular cell layer
GFAP: Glial fibrillary acidic protein
hUCB-MSCs: Human umbilical-cord-blood-derived mesenchymal stem cells
IVH: Intraventricular hemorrhage
MAPK: Mitogen-activated protein kinase
MRI: Magnetic resonance imaging
MSCs: Mesenchymal stem cells
OSC: Organotypic slice co-culture
SGZ: Subgranular zone
Tuj: Neuron-specific class III beta-tubulin
