# Supplementary figures and images for: Human UCB-MSCs treatment upon intraventricular hemorrhage contributes to attenuate hippocampal neuron loss and circuit damage through BDNF-CREB signaling
Source: Stem Cell Res Ther. 2018 Nov 21;9:326. doi: 10.1186/s13287-018-1052-5 (PMC6249960; doi:10.1186/s13287-018-1052-5)

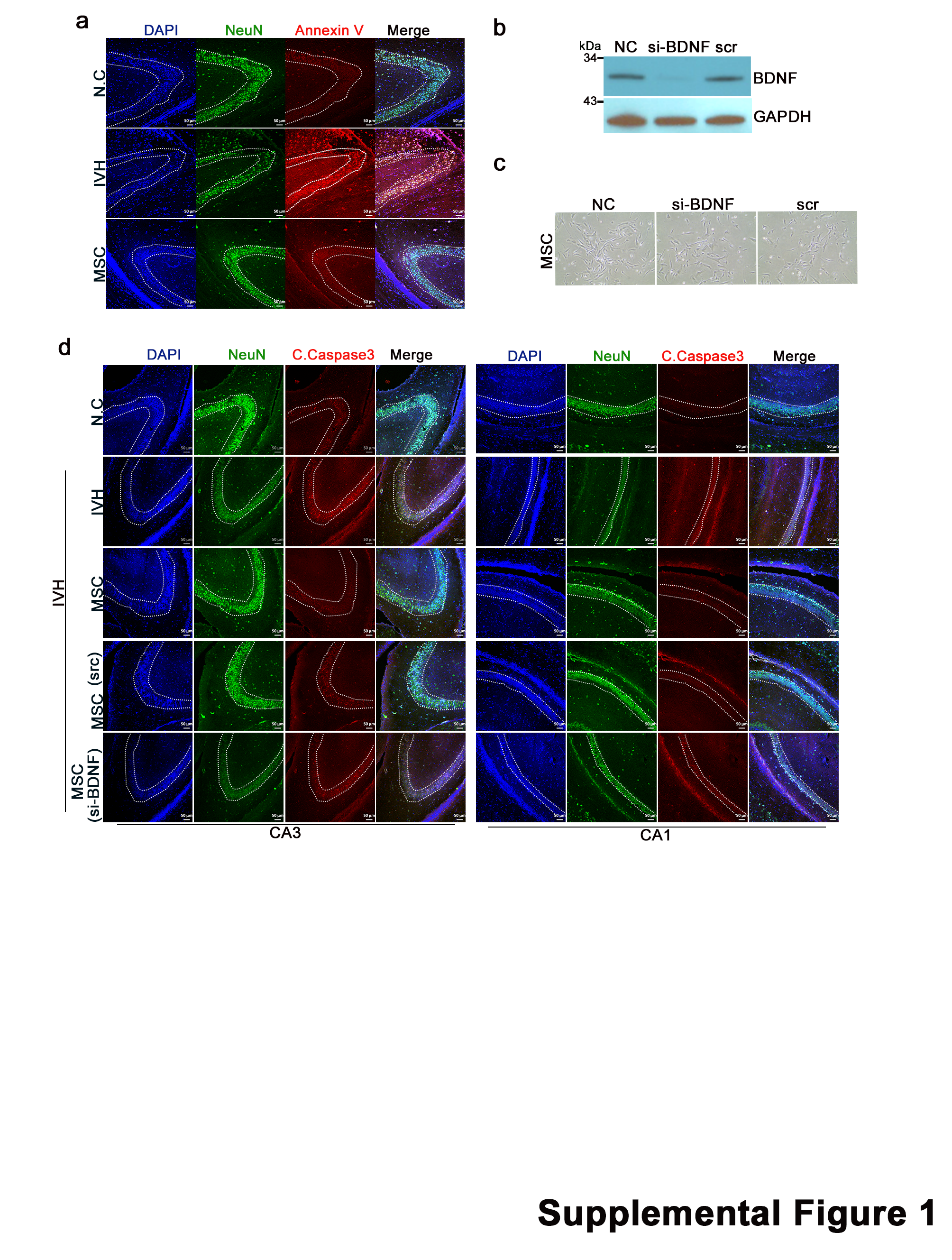

Supplement: Supplementary file 1 — Figure S1. hUCB-MSC treatment promotes neuronal survival in the hippocampus through activation of BDNF signaling. (a) Paraffin rat brain sections were stained with annexin V (red) and NeuN (green). Nuclei were counterstained with DAPI stain (blue). Images shown here are representative of at least three independent experiments. Scale bar, 50 μm (b and c) MSCs were transfected with siRNA oligonucleotides using Oligofectamine (Invitrogen, Carlsbad, CA, USA) according to the manufacturer’s instructions. From the MSCs, BDNF expression was successfully knocked-down and sustained for at least 48 h after transfection of BDNF siRNA. (d) Neuronal cell death was assessed on the paraffin section at P7. Brain section was stained with cleaved caspase 3 (red) and NeuN (green). Nuclei were counterstained with DAPI stain (blue). Images shown here are representative of at least three independent experiments. Scale bar, 50 μm. (TIF 8489 kb) [file 13287_2018_1052_MOESM1_ESM.tif]

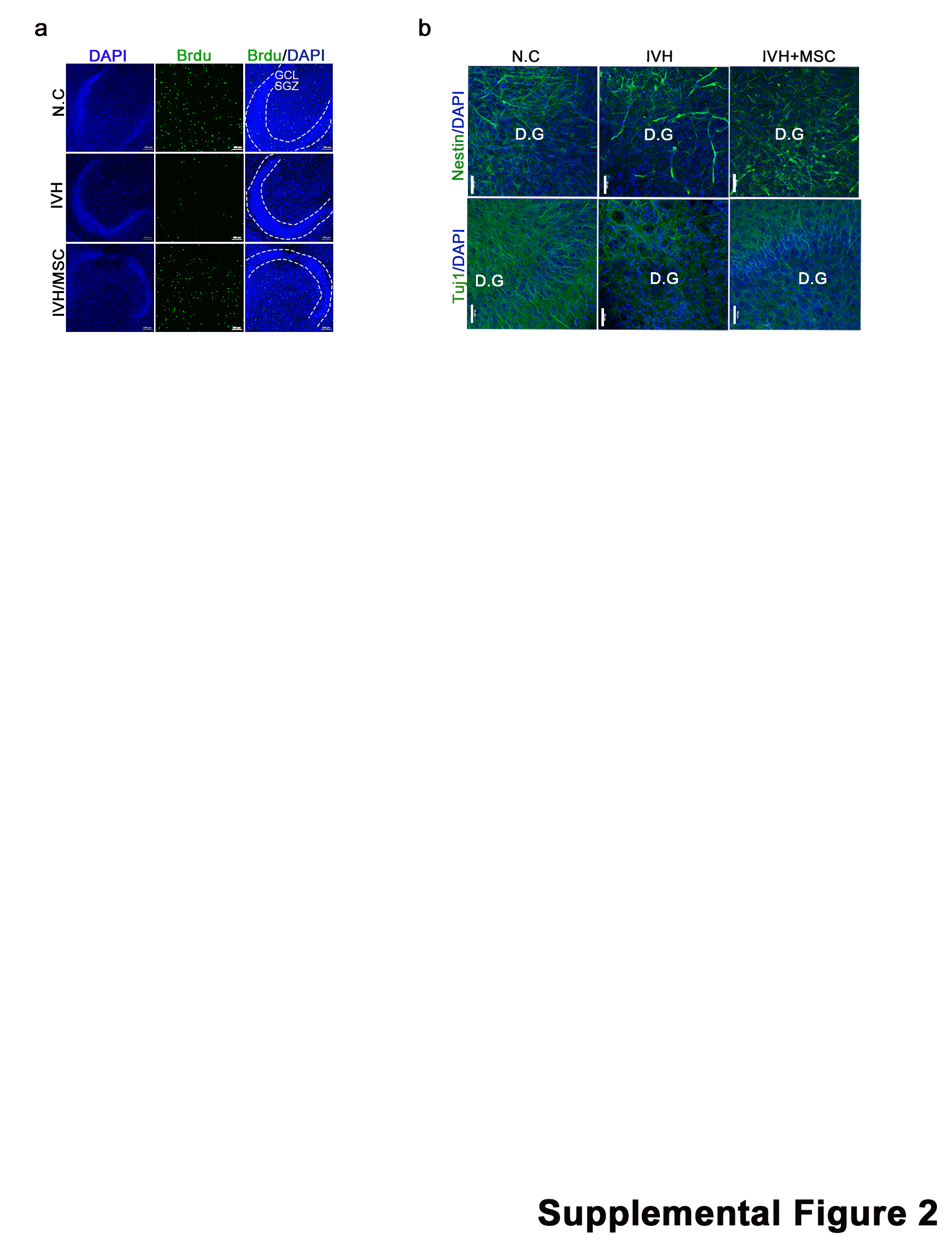

Supplement: Supplementary file 2 — Figure S2. hUCB-MSC treatment enhanced neurogenesis in the hippocampus after neuron loss. (a) Hippocampal slice culture was treated with BrdU (10μg/ml) at DIV7. The slice was fixed with 4% PFA at DIV9 and stained with anti-BrdU antibody (green). Nuclei were counterstained with DAPI stain (blue). Scale bar, 50 μm. (b) After severe IVH and transplantation of hUCB-MSCs, P7 rat models were analyzed with an entorhinal-hippocampus organotypic slice co-culture. Slice culture was fixed and stained with the neural stem cell marker nestin (green) and the neuron marker TUJ1 (green) at DIV7. Nuclei were counterstained with DAPI stain (blue). Images shown here are representative of at least three independent experiments. Scale bar, 50 μm. (TIF 3765 kb) [file 13287_2018_1052_MOESM2_ESM.tif]

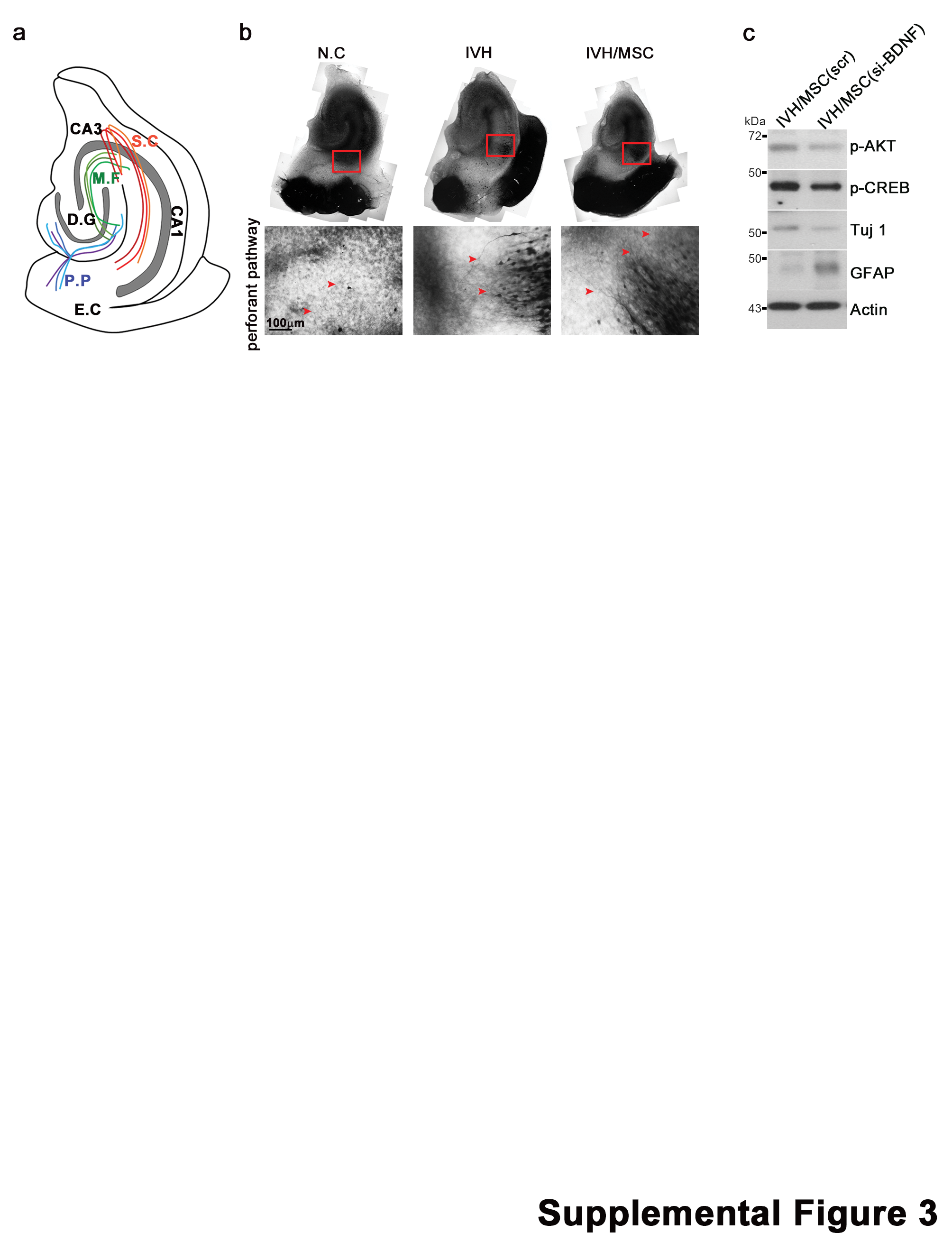

Supplement: Supplementary file 3 — Figure S3. hUCB-MSC treatment contributes to the recovery of IVH injury-mediated lesions in the hippocampal trisynaptic circuit. (a) Schematic diagram of hippocampal trisynaptic circuit. The perforant path is the connectional route from the entortinal cortex to the dentate gyrus. Signal information from the dentate gyrus projects along the mossy fiber to the cornu ammonis area 3 (CA3). Axons from CA3 project to area CA1 pyramidal neurons via Schaffer collateral fibers. (b) The hippocampal slices of IVH/MSC rat models were cultured at P7. The anterograde axonal tracer biocytin was placed on the entorhinal cortex at DIV8 and fixed with 4% PFA. Biocytin was visualized using the ABC-DAB method. Red arrows indicate the perforant fibers. The square boxed area is enlarged in the bottom panel. Images shown here are representative of at least three independent experiments. Scale bar, 100 μm. (c) Rat hippocampi were dissected and lysed at P7 in IVH/MSCs rat models. Lysates were subjected to IB with the indicated antibodies. Images shown here are representative of at least three independent experiments. (TIF 3251 kb) [file 13287_2018_1052_MOESM3_ESM.tif]
